# White matter hyperintensity burden in young patent foramen ovale patients and its correlation with migraine

**DOI:** 10.3389/fneur.2025.1737566

**Published:** 2026-01-02

**Authors:** Yuxuan Li, Yangyingqiu Liu, Jinfeng Cao, Qun Shang, Wenjing Zheng, Jiaqi Li, Xinying Shi, Jiaxiang Xin, Wei Zhao, Ye Meng, Bo Li, Xin Luo

**Affiliations:** 1 School of Medical Imaging, Shandong Second Medical University, Weifang, China; 2 Department of Radiology, Zibo Central Hospital, Zibo, China; 3 Department of Imaging, Leling People's Hospital, Dezhou, China; 4 School of Medical Imaging, Binzhou Medical University, Yantai, China; 5 MR Research Collaboration, Siemens Healthineers Ltd., Shanghai, China; 6 Department of Ultrasound, Zibo Central Hospital, Zibo, China; 7 Department of Neurology, Zibo Central Hospital, Zibo, China; 8 Department of Cardiology, Zibo Central Hospital, Zibo, China

**Keywords:** cryptogenic stroke, migraine, patent foramen ovale, right-to-left shunt, white matter hyperintensity

## Abstract

**Objective:**

Patent foramen ovale (PFO) may play an important role in the progression of white matter hyperintensity (WMH) through paradoxical embolism-induced chronic cerebral microcirculatory dysfunction. WMH is closely associated with the pathogenesis of migraine. This study aims to investigate the burden of WMH in young patients with PFO and their potential association with migraine.

**Methods:**

A retrospective analysis was conducted involving 47 young patients with PFO (PFO group) and 50 healthy controls (HC group). The Headache Impact Test-6 (HIT-6), Migraine Disability Assessment (MIDAS), and Visual Analogue Scale (VAS) scores were recorded to assess the severity of migraine. Conventional MRI was utilized to evaluate periventricular hyperintensity (PVH) and deep white matter hyperintensity (DWMH) grades, and the differences were compared between groups. Correlations among laboratory indicators, right-to-left shunt (RLS) grade, PFO diameter, WMH burden, and migraine severity were examined in PFO patients.

**Results:**

The PVH and DWMH scores were significantly higher in the PFO group than in HC (*p* < 0.001). PFO diameter and RLS grade both showed positive correlations with PVH, DWMH, VAS, MIDAS and HIT-6 scores (*p* < 0.05). The grades of PVH and DWMH were significantly positively correlated with the HIT-6, VAS, and MIDAS scores (*p* < 0.001). PFO diameter was an independent predictor of PVH and DWMH grades (*p* < 0.001).

**Conclusion:**

Young PFO patients exhibit an increased WMH burden, and PFO diameter serves as an independent risk factor for WMH grade. These findings suggest that PFO may contribute to the development of WMH in young individuals, potentially through mechanisms involving paradoxical embolism and chronic cerebral hypoperfusion. In young patients with PFO and migraine, monitoring WMH burden via neuroimaging may provide insights for future personalized management strategies.

## 1 Introduction

Patent foramen ovale (PFO) is the primary cause of right-to-left shunt (RLS), which is present in approximately 25–30% of the general population, with a notable increase to about 40% among individuals with cryptogenic stroke (CS) (1–3).

White matter hyperintensities (WMH) are areas of elevated signal intensity observed on brain magnetic resonance imaging (MRI) fluid-attenuated inversion recovery (FLAIR) sequences. They are associated with an elevated risk of future stroke, increased stroke severity, cognitive decline, and mortality, and have been identified as predictors of stroke, cognitive decline, and depression (4, 5).

The PFO facilitates a pathological mechanism for paradoxical embolism, wherein emboli originating from the venous system gain access to the arterial circulation. Current evidence suggests a correlation between PFO-related paradoxical embolism and an elevated risk of WMH (6). Additionally, research has demonstrated that cerebral microinfarcts in specific parts of the brain are linked to the presence of PFO, thereby reinforcing the association between PFO-related microembolic events and damage to brain tissue (6).

The WMH is recognized as a biomarker for chronic migraine (7). The likelihood of developing WMH in individuals with migraine is two to four times greater than in the general population (8), and this correlation may be significantly associated with the RLS (9). The proposed underlying mechanism suggests that RLS may permit microemboli or vasoactive substances from the venous system to circumvent the pulmonary circulation’s filtration function, thereby entering the cerebral arterial circulation directly. This process could potentially damage cerebral white matter, and subsequently result in the formation of WMH (9).

The RLS grade is primarily used to evaluate the severity of RLS (10), and PFO diameter is a crucial factor in assessing its severity and clinical implications (11). Although the PFO diameter and RLS are associated with WMH occurrence (12), no clear evidence indicates that PFO diameter or RLS severity directly leads to WMH aggravation. The underlying pathophysiological mechanism necessitates further investigation to validate the hypothesis that PFO facilitates the entry of microemboli or vasoactive substances into the brain, triggering chronic microcirculatory dysfunction, which in turn leads to the formation and progression of WMH.

Currently, there is a lack of systematic research on the quantitative relationship among PFO diameter, RLS grade, WMH burden, and migraine severity in young PFO patients. Therefore, this study investigates young PFO patients to minimize age-related confounding effects. By distinguishing and grading WMH in different locations, include periventricular and deep white matter, it aims to elucidate the quantitative relationships among PFO diameter, RLS grade, WMH burden, and migraine severity.

## 2 Materials and methods

### 2.1 Patient population

This study received approval from the Ethics Committee of Zibo Central Hospital (Approval No. 2024219). As a retrospective study, it was exempted from informed consent and conducted in accordance with the Declaration of Helsinki and relevant regulations.

**Figure 1:**
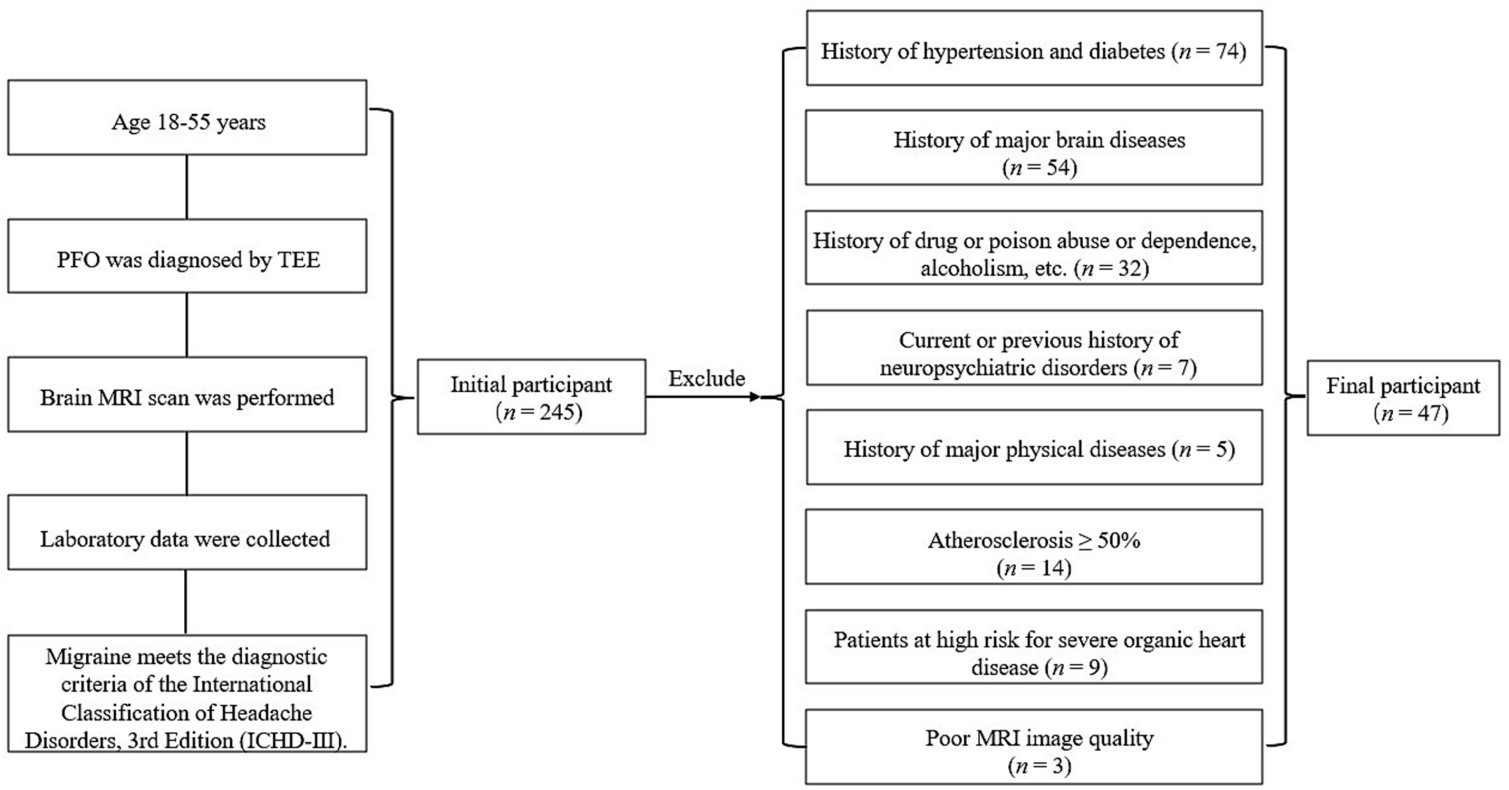
Flowchart of patient enrollment.

A total of 47 young patients diagnosed with PFO at Zibo Central Hospital (PFO group) between January 2022 and February 2024 were retrospectively enrolled (Figure 1).

The inclusion criteria for the PFO group were as follows: (1) Diagnosis of PFO via transesophageal echocardiography (TEE); (2) Age between 18 and 55 years (13), irrespective of sex; (3) Completion of cranial MRI scanning; (4) Availability of laboratory data; (5) A documented history of migraines that aligns with the diagnostic criteria outlined in the International Classification of Headache Disorders, 3rd edition (ICHD-III) (14). The exclusion criteria included: (1) A history of hypertension or diabetes mellitus; (2) A history of major cerebrovascular diseases, such as stroke, intracranial hemorrhage, brain tumors, multiple sclerosis, or significant traumatic brain injury; (3) A history of substance abuse, alcoholism, or dependence; (4) A history of neuropsychiatric disorders or a family history of such conditions; (5) A history of major systemic diseases, including malignancy, uremia, autoimmune diseases, or thyroid/parathyroid dysfunction; (6) Significant atherosclerosis, defined as stenosis of 50% or greater, as detected by CT angiography of the head and neck or color Doppler ultrasound; (7) Severe organic heart disease or other high-risk conditions for cardioembolism; (8) Poor-quality MRI images.

Healthy controls (HC group) were retrospectively selected from individuals who underwent brain MRI scanning during the same period as part of routine health examinations. The MRI scanning protocols for the HC group were matched with those of the PFO group to ensure consistency in the assessment of WMH. The control group was screened to adhere to the same exclusion criteria as the PFO group, ensuring the absence of significant cerebrovascular diseases and specified systemic conditions. Ultimately, 50 healthy controls, matched for age and sex, were included in the study.

### 2.2 Laboratory test indicators

Laboratory test indicators assessed for patients in the PFO group encompassed brain natriuretic peptide, fibrinogen, C-reactive protein, mean platelet volume, homocysteine, lipoprotein(a), total cholesterol, fasting blood glucose, serum creatinine, serum uric acid, and serum urea. All laboratory indicators were collected within 7 days preceding the MRI examination.

### 2.3 Color ultrasound data collection

A contrast transesophageal echocardiography (cTEE) procedure was conducted using a Philips EPIQ CVx (Philips Healthcare, MA, United States) by an experienced sonographer (W. Z., with 7 years of ultrasound diagnostic experience). The PFO diameter was measured using an electronic caliper, and the maximum opening of the PFO was recorded during the end-diastolic phase. Absolute contraindications included dysphagia, esophageal pathology, or recent surgery. Relative contraindications, such as esophageal varices or active upper gastrointestinal bleeding, necessitated a pre-procedural risk assessment (15).

Patients were positioned in the left lateral decubitus orientation for the purpose of conducting contrast echocardiography. An agitated saline solution was administered via the left antecubital vein. The presence, timing, and grading of RLS were evaluated during both resting conditions and the Valsalva maneuver, as confirmed by TTE (Transthoracic Echocardiograph). The severity of RLS was classified based on the number of microbubbles detected in the left atrium during TTE: Grade 1 (minimal) was characterized by 1–10 bubbles; Grade 2 (moderate) by 11–25 bubbles; and Grade 3 by more than 25 bubbles or complete left atrial opacification (10). RLS was further categorized as either persistent, occurring at rest without the Valsalva maneuver, or provoked, occurring exclusively during the Valsalva maneuver. Additionally, during TTE assessment, the presence of an Atrial Septal Aneurysm (ASA) was documented, defined as a protrusion of the septal base measuring ≥10 mm beyond the atrial septal plane or a total excursion of ≥15 mm throughout the cardiac cycle (16).

### 2.4 MRI data acquisition

All participants underwent brain magnetic resonance imaging (MRI) using a 3.0-Tesla clinical magnetic resonance scanner (MAGNETOM Skyra; Siemens, Erlangen, Germany) equipped with a 20-channel phased-array head surface coil. Routine MRI protocols included T1-weighted imaging (T1WI) fluid-attenuated inversion recovery (FLAIR) and T2-weighted imaging turbo spin-echo (T2WI TSE), and T2WI-FLAIR sequence scans, covering the whole brain. Scanning sequence parameters are presented in Supplementary Table S1.

### 2.5 WMH grade

The location of WMH was categorized as either periventricular hyperintensity (PVH) or deep white matter hyperintensity (DWMH). The grading scale for each is typically from 0 to 3, based on visual rating scales like the Fazekas scale (17). Grade 0: Absence of both PVH and DWMH. Grade 1: PVH presents as “caps” or pencil-thin lining; DWMH as punctate foci. Grade 2: PVH forms a smooth “halo”; DWMH shows beginning confluence of foci. Grade 3: PVH appears irregular and extends into deep white matter; DWMH appears as large confluent areas. Two neuroradiologists, one with 20 years of experience (Q. S.) and another with 10 years of experience (Y. L.), who were blinded to clinical information including age, sex, and group allocation, independently graded the WMH for all participants. Any discrepancies were resolved through consensus discussion.

### 2.6 Headache impact test-6 (HIT-6), visual analog scale (VAS) and migraine disability assessment (MIDAS) scoring

Migraine severity was evaluated using the HIT-6, VAS, and MIDAS scores, which assesses headache-specific symptoms and the functional impairment associated with headache disorders. This evaluation was performed by a neurologist (Y. M.) with 10 years of experience.

### 2.7 Statistical analysis

Statistical analyses were carried out using the R software package^1^ and the Statistical Package for Social Sciences version 27.0 (IBM Corp., Armonk, NY, United States). The inter-rater reliability for PVH and DWMH grading was assessed utilizing the Kappa statistic. Categorical data were presented as frequencies (percentages) and compared using chi-square tests. Continuous data were initially tested for normality. For data that did not follow a normal distribution, differences were represented as medians with interquartile ranges and analyzed using nonparametric statistical tests. Partial correlation analysis, adjusted for age and sex, was employed to investigate the associations between PFO diameter, WMH grade, RLS grade, RLS classification (persistent/provoked), presence of ASA, migraine severity (measured by HIT-6, VAS and MIDAS), and various laboratory indicators. Ordinal multinomial logistic regression analysis was conducted to assess the impact of PFO diameter and LS grade on WMH grade. *p* < 0.05 was considered indicative of statistical significance.

## 3 Results

### 3.1 Clinical data and laboratory indicators

The study included a total of 47 patients with PFO (comprising 17 men and 30 women, aged 18–55 years). The HC group consisted of 50 participants (comprising 17 men and 33 women, aged 18–55 years). Comparative analysis between the two groups showed no significant differences in terms of sex and age (*p* = 0.823 and 0.441, respectively). Provoked RLS was identified in 29 patients (61.7%), whereas persistent RLS was observed in 18 patients (38.3%). An ASA was detected in 9 patients with PFO, accounting for 19.1% of the cohort. Within the PFO group, the HIT-6 scores ranged from 36 to 78, with a median score of 44, the VAS scores ranged from 0 to 10, with a median score of 2.5, the MIDAS scores ranged from 0 to 540, with a median score of 37. The mean disease duration among migraine patients was 2.6 years. The frequency of migraine attacks was 2.0 episodes per month (Table 1). The laboratory data characteristics of the PFO group are detailed in Supplementary Table S2.

**Table 1:** Characteristics of PFO and HC groups.

Characteristic	PFO group (*n* = 47)		HC group (*n* = 50)	*χ*^2^/*T*	*p* value
Sex (male)	17(36.2%)		17(34.0%)	0.05	0.823
Age (year)	37.00 ± 20.00		38.00 ± 13.00	−0.774	0.441
PFO diameter (mm)	1.30(0.60)		–		
RLS	Grade 1	3(6.4%)	–		
	Grade 2	11(23.4%)	–		
	Grade 3	33(70.2%)	–		
RLS type (provocative)	29(61.7%)		–		
ASA	9(19.1%)		–		
HTI-6	44.0(16.0)		–		
MIDAS	37(80.0)		–		
VAS	2.5(6.0)		–		
Mean disease duration (year)	2.6(9.0)		–		
Attack frequency (episodes/month)	2.0(4.0)		–		

HC, healthy control; PFO, patent foramen ovale; RLS, right-to-left shunt; ASA, atrial septal aneurysm; HTI-6, headache impact test-6; MIDAS, migraine disability assessment; VAS, visual analog scale.

### 3.2 WMH grade consistency test

The Kappa test results indicated excellent inter-rater reliability in the grading of WMH between the two neuroradiologist, with Kappa coefficients of 0.983 for PVH and 0.910 for DWMH, both with *p* < 0.001.

### 3.3 WMH grade group comparison

**Figure 2:**
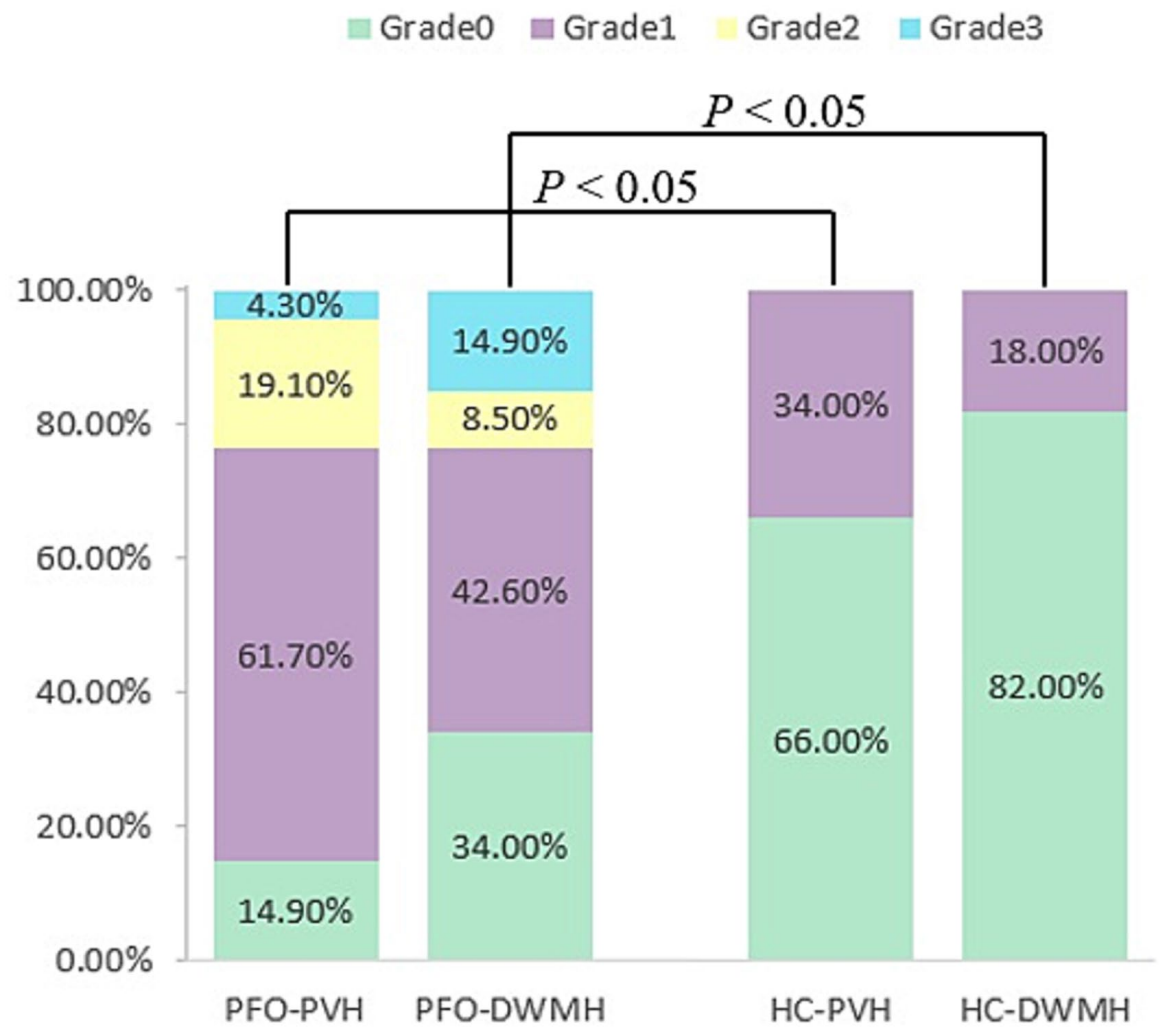
Column diagram of WMH grade between PFO and HC groups. It shows the distribution of PVH and DWMH grade in the PFO and the HC groups. DWMH, deep white matter hyperintensity; HC, healthy control; PFO, patent foramen ovale; PVH, periventricular hyperintensity.

**Figure 3:**
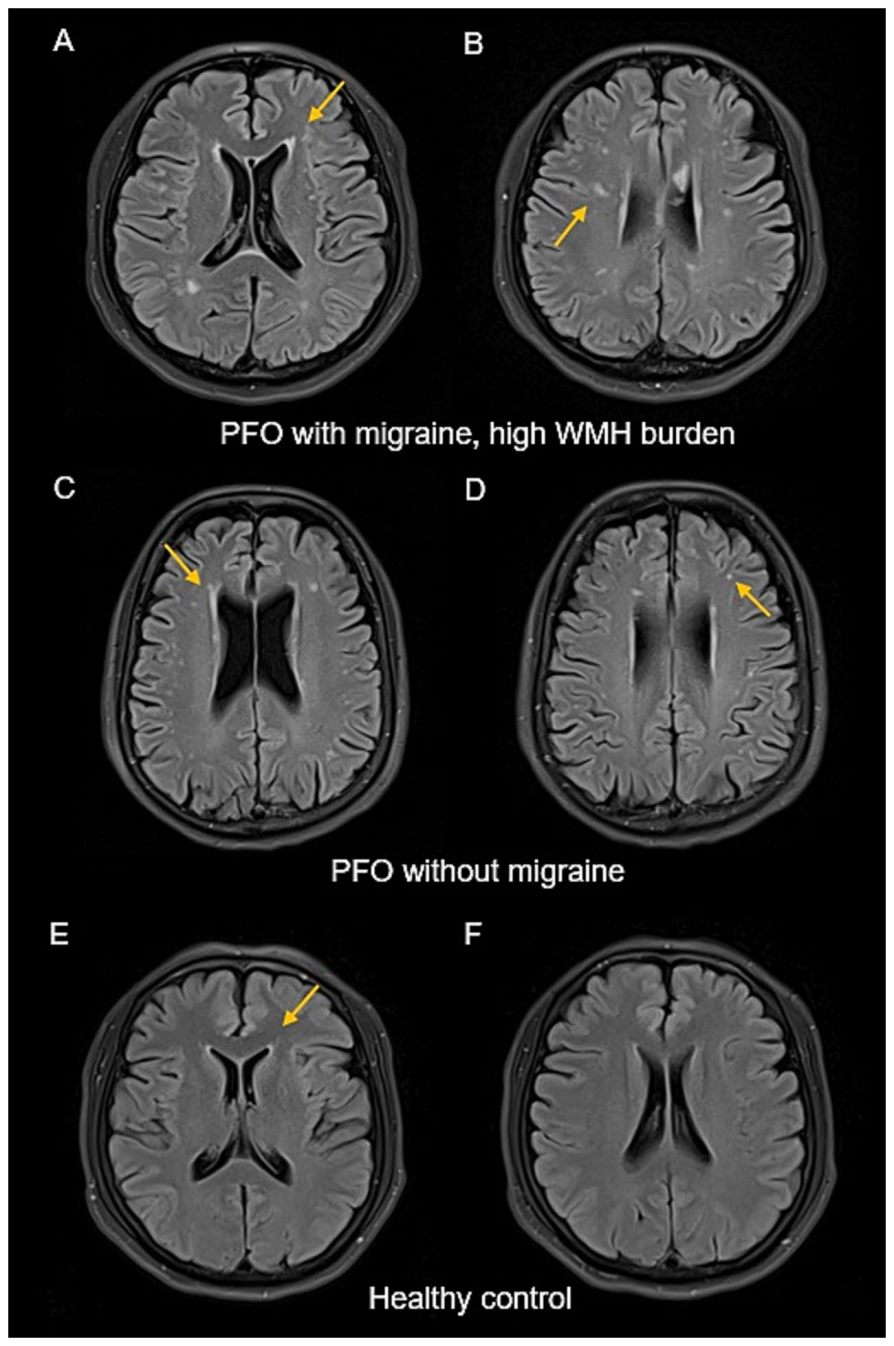
Representative images of WMH. FLAIR image of patient 1 with PFO: female, 50 years old, migraine, PFO diameter 3.3 mm, PVH Grade 2 **(A)** and DWMH Grade 3 **(B)**. FLAIR image of patient 2 with PFO: male, 47 years old, without migraine, PFO diameter 2.0 mm, PVH Grade 2 **(C)** and DWMH Grade 1 **(D)**. FLAIR image of HC: female, 49 years old, PVH Grade 1 **(E)** and DWMH Grade 0 **(F)**. WMH, white matter hyperintensity; DWMH, deep white matter hyperintensity; HC, healthy control; PFO, patent foramen ovale; PVH, periventricular hyperintensity.

Comparative analysis of WMH grades between the PFO and HC groups revealed significantly higher grades for both PVH and DWMH in the PFO group (*p* < 0.001 for both), as shown in Table 2 and Figure 2. Representative images of WMH were shown in Figure 3.

**Table 2:** Comparison of WMH grades between PFO and HC groups.

Type	Grade	PFO group (*n* = 47)	HC group (*n* = 50)	*χ* ^2^	*p-*value
PVH	0	7(14.9%)	33(66.0%)	30.967	<0.001
	1	29(61.7%)	17(34.0%)		
	2	9(19.1%)	0		
	3	2(4.3%)	0		
DWMH	0	16(34.0%)	41(82.0%)	26.069	<0.001
	1	20(42.6%)	9(18.0%)		
	2	4(8.5%)	0		
	3	7(14.9%)	0		

DWMH, deep white matter hyperintensity; PVH, periventricular hyperintensity; WMH, white matter hyperintensity.

### 3.4 Correlation of PFO diameter with laboratory indicators, HIT-6 score, VAS score, MIDAS score, RLS grade and WMH grade

Partial correlation analysis, controlling for sex and age, demonstrated a positive correlation between the PFO diameter and PVH (*r* = 0.539, *p* < 0.001), DWMH (*r* = 0.550, *p* < 0.001), HIT-6 score (*r* = 0.751, *p* < 0.001), VAS score (*r* = 0.577, *p* < 0.001), MIDAS score (*r* = 0.751, *p* < 0.001), and RLS grades (*r* = 0.296, *p* = 0.048). Furthermore, PVH (*r* = 0.430, *p* = 0.003), DWMH (*r* = 0.365, *p* = 0.014), VAS score (*r* = 0.480, *p* = 0.001), MIDAS score (*r* = 0.453, *p* = 0.002), and HIT-6 score (*r* = 0.452, *p* = 0.002) were positively correlated with RLS grade. The PVH grade was found to have a positive correlation with HIT-6 score (*r* = 0.643, *p* < 0.001), VAS score (*r* = 0.764, *p* < 0.001), and MIDAS score (*r* = 0.654, *p* < 0.001). The DWMH grade was found to have a positive correlation with HIT-6 score (*r* = 0.712, *p* < 0.001), VAS score (*r* = 0.817, *p* < 0.001), and MIDAS score (*r* = 0.675, *p* < 0.001).

**Figure 4:**
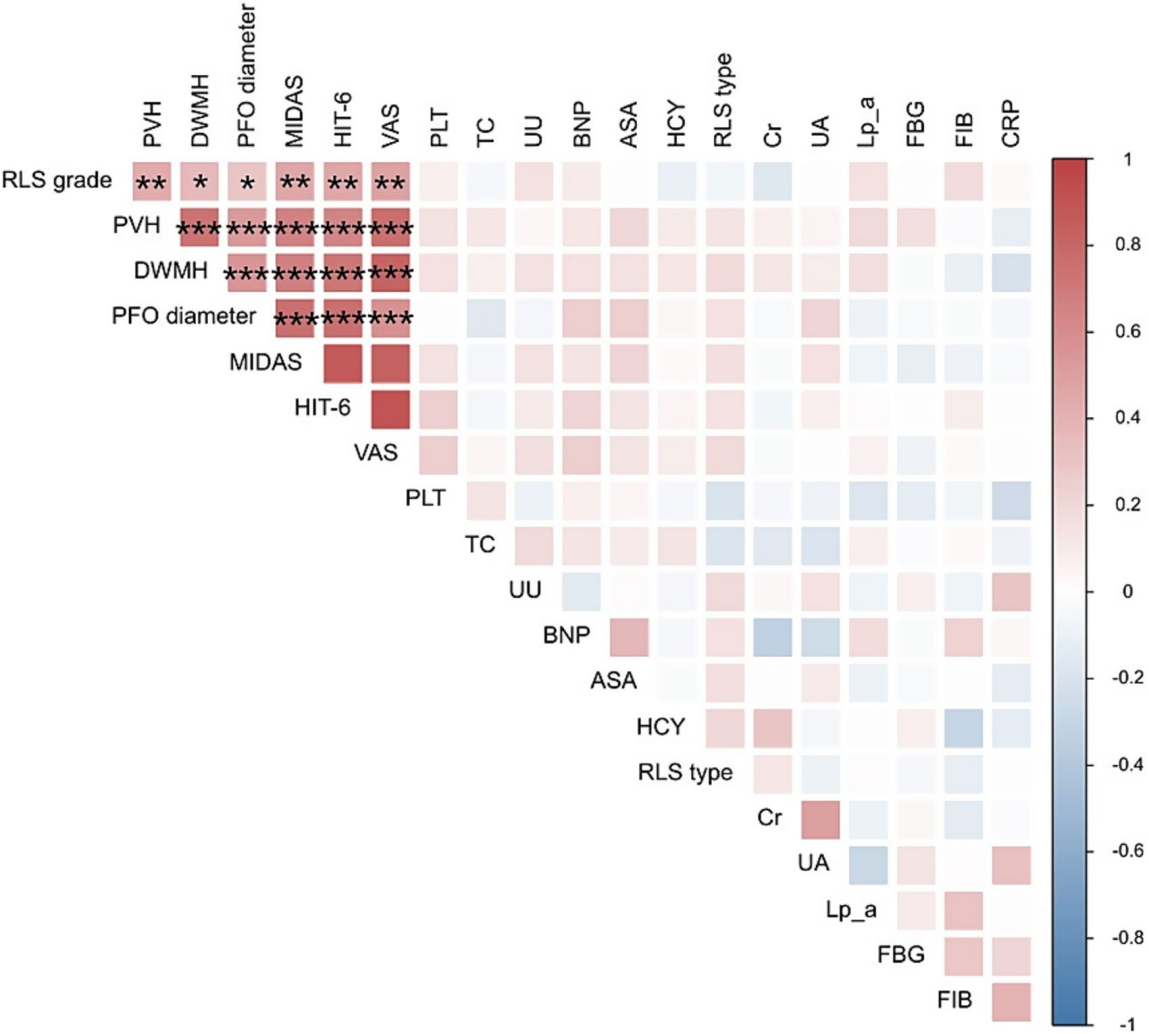
Heat map depicting the correlation among PFO diameters, WMH grades, RLS grade, HTI-6 scores, VAS scores, MIDAS scores, and laboratory indexes. The color bar on the right and the symbols in the figure display correlations. BNP, brain natriuretic peptide; FIB, fibrinogen; Cr, serum creatinine; CRP, c-reactive protein; DWMH, deep white matter hyperintensity; FBG, fasting blood glucose; HCY, homocysteine; Lp_a, lipoprotein(a); MPV, mean platelet volume; PFO, patent foramen ovale; PVH, periventricular hyperintensity; RLS, right-to-left shunt; TC, total cholesterol; UA, serum uric acid; UU, serum urea; HTI-6, headache impact test-6; MIDAS, migraine disability assessment; VAS, Visual Analogue Scale. ^*^p < 0.05, ^**^p < 0.01, and ^***^p < 0.001.

Figure 4 presents a heatmap illustrating the correlations among PFO diameter, RLS grade, HIT-6 scores, VAS scores, MIDAS scores, laboratory indicators, and WMH grades.

However, neither the type of RLS (provoked vs. persistent) nor the presence of ASA exhibited statistically significant correlations with WMH grades, HIT-6 scores, VAS scores, or MIDAS scores (*p* > 0.05).

### 3.5 Ordinal multinomial logistic regression analysis of the impact of PFO diameter and RLS grade on PVH and DWMH grades

The ordinal multinomial logistic regression analysis assessing the impact of PFO diameter on PVH and DWMH grades indicated that PFO diameter serves as an independent influencing factor for both PVH and DWMH grades (*p* < 0.001) (Table 3).

**Table 3:** Ordered multiple logistic regression analysis of the effect of PFO diameter on PVH and DWMH grades.

Variable	*B*	95% CI		*p-*value
		Lower limit	Upper limit	
PVH	2.247	1.031	3.464	<0.001
DWMH	1.841	0.749	2.933	<0.001

CI, confidential interval; DWMH, deep white matter hyperintensity; PFO, patent foramen ovale; PVH, periventricular hyperintensity.

## 4 Discussion

This study concentrated on young patients with PFO, identifying a significantly increased burden of WMH. Subsequent analysis demonstrated that the PFO diameter was strongly correlated with the severity of both PVH and DWMH, as well as with the grade of RLS. Notably, multivariate analysis confirmed that the PFO diameter serves as an independent risk factor influencing the severity of PVH and DWMH.

The findings suggest that PFO is a risk factor not only for stroke but also for an extensive WMH burden in the brain (6). However, previous studies have not consistently established an association between PFO and WMH. This inconsistency may be attributed to the greater impact of PFO on stroke risk in younger patients, whereas studies such as that by Di Tullio et al. (18) involved participants with a relatively high mean age (68.4 ± 9.4 years). Advanced age itself is a significant risk factor for cerebrovascular disease and WMH, and its substantial confounding effect may obscure the relatively weaker independent contribution of PFO. In this study, we focused on young individuals to minimize age-related confounding factors in stroke risk and to investigate the association between PFO and the severity of WMH.

The PFO is recognized as a risk factor for paradoxical embolism and stroke, serving as an anatomical conduit for emboli to reach the brain and potentially cause cerebrovascular events (19, 20). WMH is a prevalent neuroimaging marker indicative of brain health (21). The Fazekas scoring system categorizes WMH into two regions: PVH and DWMH, assessing the severity of each separately. PVH is characterized by gliosis, myelin pallor, and demyelination, whereas DWMH is more heterogeneous, with greater axonal loss, vacuolation, and arteriolosclerosis (22). Prior research has associated PFO with white matter lesions (23, 24), primarily located in the deep white matter and appearing punctate (25). Our findings demonstrate a positive correlation between increased PVH and DWMH burden and the PFO diameter. Regression analysis revealed that the PFO diameter is an independent predictor of both PVH and DWMH grades. This suggests that patients with larger PFO diameters may experience increased microembolic entry into the arterial circulation, impacting the cerebral microvasculature. Consequently, patients with larger PFO diameters demonstrate increased cerebral gliosis, myelin pallor, demyelination, axonal loss, vacuolation, and arteriolosclerosis.

This study also identified a positive correlation between RLS and both WMH burden and migraine severity, aligning with previous research findings (3, 12). RLS has been implicated as a potential contributing factor for stroke and migraine with aura in the presence of PFO (26). As the RLS grade escalates, the passage of small microemboli or vasoactive substances, such as serotonin, through the PFO also increases (27). These factors may precipitate migraines via cortical spreading depression (CSD) and induce cerebral white matter microvascular damage and an elevated WMH burden through direct or indirect CSD-related ischemia or neuroinflammation (28, 29). This suggests that the observed increase in WMH burden in young PFO patients may involve overlapping or interacting pathophysiological processes associated with both RLS and migraine.

The relationship between migraine and WMH has garnered substantial attention, with WMH being considered a marker of chronic migraine (7), potentially affecting cognitive function and other neurological outcomes. The migraine severity assessment indicators in this study, including the VAS, HIT-6, and MIDAS scores, demonstrated statistically significant correlations with the grades of PVH and DWMH. A substantial of neuroimaging studies has demonstrated that migraine patients are more likely to exhibit WMH compared to HC (29, 30). And a significant proportion of migraine patients (63.9%) exhibit WMH on T2WI and T2WI-FLAIR (26, 31). The pathophysiological mechanisms underlying WMH in migraine have not been fully elucidated. Possible mechanisms include: brain injury induced by metalloproteinases activated during cortical spreading depolarization, ischemic microvascular dysfunction with subsequent regional hypoperfusion, microembolization, hypercoagulable states, and endothelial dysfunction (29). Preclinical studies have demonstrated that microemboli, such as air, polystyrene microspheres, and cholesterol particles, can induce CSD (32). Smaller emboli are associated with rapid and transient CSD, accompanied by a significant reduction in cerebral blood flow (CBF) (33). This mechanism, wherein microemboli induce CSD that may progress to ischemic events, offers a biological rationale for the observed association between migraine and ischemic stroke in patients with PFO (33, 34).

This study acknowledges several limitations that suggest avenues for future research. Firstly, the small sample size and reliance on a single-source origin necessitate validation in larger cohorts. Secondly, since TTE is an invasive procedure and was not performed on the HC group, along with missing blood biomarker information, may introduce potential selection bias. Thirdly, the manual classification of WMH based on two-dimensional (2D) imaging data constrains the accuracy of quantifying WMH burden in patients with PFO. Future studies should incorporate artificial intelligence methodologies combined with three-dimensional (3D) imaging to achieve more precise volumetric measurements and standardized quantification. Lastly, as this was a retrospective study, data on the presence of migraine aura were unavailable. These data will be systematically collected and analyzed in future prospective studies.

## 5 Conclusion

In conclusion, young patients with PFO exhibit a higher burden of WMH, and the PFO diameter emerges as an independent risk factor for the severity of WMH. These findings suggest that PFO may contribute to the development of WMH in young individuals, potentially through mechanisms involving paradoxical embolism and chronic cerebral hypoperfusion. In young patients with PFO and migraine, monitoring WMH burden via neuroimaging may provide insights for future personalized management strategies.

## Data Availability

The original contributions presented in the study are included in the article/Supplementary material, further inquiries can be directed to the corresponding authors.

## Footnotes

1
http://www.R-project.org/

## References

[1] Mac Grory B Ohman EM Feng W Xian Y Yaghi S Kamel H . Advances in the management of cardioembolic stroke associated with patent foramen ovale. BMJ. (2022) 376:e063161. doi: 10.1136/bmj-2020-063161, 35140114

[2] Lechat P Mas JL Lascault G Loron PH Theard M Klimczac M . Prevalence of patent foramen ovale in patients with stroke. N Engl J Med. (1988) 318:1148–52. doi: 10.1056/NEJM198805053181802, 3362165

[3] Jiang XH Wang SB Tian Q Zhong C Zhang GL Li YJ . Right-to-left shunt and subclinical ischemic brain lesions in Chinese migraineurs: a multicentre MRI study. BMC Neurol. (2018) 18:18. doi: 10.1186/s12883-018-1022-7, 29444659 PMC5813373

[4] Duering M Biessels GJ Brodtmann A Chen C Cordonnier C de Leeuw FE . Neuroimaging standards for research into small vessel disease-advances since 2013. Lancet Neurol. (2023) 22:602–18. doi: 10.1016/S1474-4422(23)00131-X, 37236211

[5] Kuller LH Longstreth WT Jr Arnold AM Bernick C Bryan RN Beauchamp NJ. White matter hyperintensity on cranial magnetic resonance imaging: a predictor of stroke. Stroke. (2004) 35:1821–5. doi: 10.1161/01.STR.0000132193.35955.69, 15178824

[6] Badea RȘ Mihăilă-Bâldea S Ribigan A Negrilă A Grecu N Marinescu AN . PFO-spectrum disorder: two different cerebrovascular diseases in patients with PFO as detected by AI brain imaging software. Front Neurol. (2024) 15:1357348. doi: 10.3389/fneur.2024.1357348, 38440117 PMC10909929

[7] Abdalla K Alawneh KZ Al-Bdour M Abu-Salih AQ. Migraine and MRI: uncovering potential associations. Head Face Med. (2025) 21:6. doi: 10.1186/s13005-024-00478-2, 39955604 PMC11830205

[8] Huo J Zhang G Wang W Cao W Wan M Huang T . Migraine and white matter lesions: a mendelian randomization study. Sci Rep. (2023) 13:10984. doi: 10.1038/s41598-023-38182-x, 37415088 PMC10326014

[9] Iwasaki A Suzuki K Takekawa H Takashima R Suzuki A Suzuki S . The relationship between right-to-left shunt and brain white matter lesions in Japanese patients with migraine: a single center study. J Headache Pain. (2017) 18:3. doi: 10.1186/s10194-016-0714-x, 28063107 PMC5218958

[10] Gaspardone A Sgueglia GA De Santis A D'Ascoli E Iamele M Piccioni F . Predictors of residual right-to-left shunt after percutaneous suture-mediated patent fossa ovalis closure. JACC Cardiovasc Interv. (2020) 13:2112–20. doi: 10.1016/j.jcin.2020.06.004, 32972572

[11] Liu X Zhang Y Xie H Zeng H Sun J Su L . Change in patent foramen ovale height is associated with cryptogenic stroke and the construction of a morphology-based scoring system. Front Cardiovasc Med. (2022) 9:1010947. doi: 10.3389/fcvm.2022.1010947, 36518683 PMC9742367

[12] Song H Zhang S Xie Q Zhu Z Li L Zhao H. Compromised cerebrovascular reactivity related to presence of white matter hyperintensities in cryptogenic stroke with right-to-left shunts. J Stroke Cerebrovasc Dis. (2025) 34:108223. doi: 10.1016/j.jstrokecerebrovasdis.2025.108223, 39778666

[13] Tromp J Paniagua SMA Lau ES Allen NB Blaha MJ Gansevoort RT . Age dependent associations of risk factors with heart failure: pooled population based cohort study. BMJ. (2021) 372:n461. doi: 10.1136/bmj.n461, 33758001 PMC7986583

[14] Olesen J. The international classification of headache disorders: history and future perspectives. Cephalalgia. (2024) 44:3331024231214731. doi: 10.1177/03331024231214731, 38166472

[15] Khandheria BK Seward JB Tajik AJ. Transesophageal echocardiography. Mayo Clin Proc. (1994) 69:856–63. doi: 10.1016/s0025-6196(12)61788-1, 8065188

[16] Niiyama S Ueno Y Kurita N Nakajima S Kijima C Hira K . White matter lesions as a prognostic marker of recurrence in cryptogenic stroke with high-risk patent foramen ovale. J Stroke Cerebrovasc Dis. (2024) 33:108048. doi: 10.1016/j.jstrokecerebrovasdis.2024.108048, 39476743

[17] Huang WQ Lin Q Tzeng CM. Leukoaraiosis: epidemiology, imaging, risk factors, and management of age-related cerebral white matter Hyperintensities. J Stroke. (2024) 26:131–63. doi: 10.5853/jos.2023.02719, 38836265 PMC11164597

[18] Di Tullio MR Jin Z Russo C Elkind MSV Rundek T Yoshita M . Patent foramen ovale, subclinical cerebrovascular disease, and ischemic stroke in a population-based cohort. J Am Coll Cardiol. (2013) 62:35–41. doi: 10.1016/j.jacc.2013.03.064, 23644084 PMC3696432

[19] Shah AH Horlick EM Kass M Carroll JD Krasuski RA. The pathophysiology of patent foramen ovale and its related complications. Am Heart J. (2024) 277:76–92. doi: 10.1016/j.ahj.2024.08.001, 39134216

[20] Elgendy AY Saver JL Amin Z Boudoulas KD Carroll JD Elgendy IY . Proposal for updated nomenclature and classification of potential causative mechanism in patent foramen Ovale-associated stroke. JAMA Neurol. (2020) 77:878–86. doi: 10.1001/jamaneurol.2020.0458, 32282016

[21] Gwak DS Ryu WS Schellingerhout D Chung J Kim HR Jeong SW . Effects of white matter hyperintensity burden on functional outcome after mild versus moderate-to-severe ischemic stroke. Sci Rep. (2024) 14:22567. doi: 10.1038/s41598-024-71936-9, 39343768 PMC11439954

[22] Griffanti L Jenkinson M Suri S Zsoldos E Mahmood A Filippini N . Classification and characterization of periventricular and deep white matter hyperintensities on MRI: a study in older adults. NeuroImage. (2018) 170:174–81. doi: 10.1016/j.neuroimage.2017.03.024, 28315460

[23] Signoriello E Cirillo M Puoti G Signoriello G Negro A Koci E . Migraine as possible red flag of PFO presence in suspected demyelinating disease. J Neurol Sci. (2018) 390:222–6. doi: 10.1016/j.jns.2018.04.042, 29801894

[24] Purandare N Oude Voshaar RC McCollum C Jackson A Burns A. Paradoxical embolisation and cerebral white matter lesions in dementia. Br J Radiol. (2008) 81:30–4. doi: 10.1259/bjr/90498392, 17998278

[25] Severa G Cortese R Covelli A Battaglini M Zhang J Lucchetti L . White matter lesion characteristics on MRI can differentiate multiple sclerosis from patent foramen ovale. J Neurol Sci. (2021) 429:118301. doi: 10.1016/j.jns.2021.118301

[26] Borończyk M Zduńska A Węgrzynek-Gallina J Grodzka O Lasek-Bal A Domitrz I. Migraine and stroke: correlation, coexistence, dependence – a modern perspective. J Headache Pain. (2025) 26:39. doi: 10.1186/s10194-025-01973-w, 39979846 PMC11844069

[27] Kim JW Kim SJ Yoon CW Park CH Kang KW Kim SK . Association between the amount of right-to-left shunt and infarct patterns in patients with cryptogenic embolic stroke: a transcranial Doppler study. Int J Stroke. (2013) 8:657–62. doi: 10.1111/j.1747-4949.2012.00846.x, 22812924

[28] Liu K Wang BZ Hao Y Song S Pan M. The correlation between migraine and patent foramen Ovale. Front Neurol. (2020) 11:543485. doi: 10.3389/fneur.2020.543485, 33335507 PMC7736411

[29] Al-Hashel JY Alroughani R Gad K Al-Sarraf L Ahmed SF. Risk factors of white matter hyperintensities in migraine patients. BMC Neurol. (2022) 22:159. doi: 10.1186/s12883-022-02680-8, 35488255 PMC9052543

[30] Kruit MC van Buchem MA Hofman PA Bakkers JT Terwindt GM Ferrari MD . Migraine as a risk factor for subclinical brain lesions. JAMA. (2004) 291:427–34. doi: 10.1001/jama.291.4.427, 14747499

[31] Zhang W Cheng Z Fu F Zhan Z. Prevalence and clinical characteristics of white matter hyperintensities in migraine: a meta-analysis. Neuroimage Clin. (2023) 37:103312. doi: 10.1016/j.nicl.2023.103312, 36610309 PMC9827384

[32] Dalkara T Nozari A Moskowitz MA. Migraine aura pathophysiology: the role of blood vessels and microembolisation. Lancet Neurol. (2010) 9:309–17. doi: 10.1016/S14744422(09)70358-8 20170844 PMC2921876

[33] Sacco S Harriott AM Ayata C Ornello R Bagur R Jimenez-Ruiz A . Microembolism and other links between migraine and stroke: clinical and pathophysiologic update. Neurology. (2023) 100:716–26. doi: 10.1212/WNL.0000000000201699, 36522158 PMC10103117

[34] Dönmez-Demir B Yemisci M Kılıç K Gürsoy-Özdemir Y Söylemezoğlu F Moskowitz M . Microembolism of single cortical arterioles can induce spreading depression and ischemic injury; a potential trigger for migraine and related MRI lesions. Brain Res. (2018) 1679:84–90. doi: 10.1016/j.brainres.2017.11.023, 29183666

