# Supplementary material for: White matter hyperintensity burden in young patent foramen ovale patients and its correlation with migraine
Source: Front Neurol. 2026 Jan 2;16:1737566. doi: 10.3389/fneur.2025.1737566 (PMC12807939; doi:10.3389/fneur.2025.1737566)
Supplement: Supplementary file 1 [file Table_1.docx]

**Table S1.** MRI protocols

| Sequence | T1WI-FLAIR | T2WI TSE | T2WI-FLAIR |
| --- | --- | --- | --- |
| TR (ms) | 2000 | 4000 | 9000 |
| TE (ms) | 36 | 99 | 86 |
| TI (ms) | 893 | – | 2499 |
| Slice thickness (mm) | 5.0 | 5.0 | 5.0 |
| Averages | 2.0 | 2.0 | 1.0 |
| FOV | 220×200 | 220×200 | 220×200 |
| Matrix | 320×203 | 384×261 | 320×203 |
| Acquisition time (s) | 108 | 54 | 108 |

FLAIR, Fluid-attenuated inversion recovery; FOV, field of view; T1WI, T1-weighted imaging; T2WI, T2-weighted imaging; TE, echo time; TI, inversion time; TR, repetition time; TSE,turbo spin–echo.

**Table S2.** Laboratory data of the PFO group

|  | PFO group (*n*=47) |
| --- | --- |
| BNP (pg/mL) | 30.50(38.30) |
| FIB (g/L) | 2.70±0.73 |
| CRP (mg/L) | 0(0.67) |
| MPV (fL) | 10.00±1.20 |
| HCY (μmol/L) | 13.40(6.00) |
| Lp_a (mg/L) | 90.00(204.40) |
| TC (mmol/L) | 4.23±0.82 |
| FBG (mmol/L) | 5.09±0.54 |
| Cr (μmol/L) | 63.00±18.00 |
| UA (μmol/L) | 289.00±97.00 |
| UU (mmol/L) | 4.4(1.70) |

BNP, Brain natriuretic peptide; FIB, fibrinogen; Cr, serum creatinine; CRP, C-reactive protein; FBG, fasting blood glucose; HC, healthy control; HCY, homocysteine; Lp_a, lipoprotein(a); MPV, mean platelet volume; PFO, patent foramen ovale; TC, total cholesterol; UA, serum uric acid; UU, serum urea.
